# Predicting the impact of genotype-by-genotype interaction on the purebred–crossbred genetic correlation from phenotype and genotype marker data of parental lines

**DOI:** 10.1186/s12711-022-00773-z

**Published:** 2023-01-13

**Authors:** Pascal Duenk, Yvonne C. J. Wientjes, Piter Bijma, Maja W. Iversen, Marcos S. Lopes, Mario P. L. Calus

**Affiliations:** 1 Wageningen University & Research, P.O. Box 338, 6700 AH Wageningen, The Netherlands; 2 Norsvin SA, Storhamargata 44, 2317 Hamar, Norway; 3 Topigs Norsvin Research Center, P.O. Box 43, 6640 AA Beuningen, The Netherlands; 4 Topigs Norsvin, Curitiba, 80420-210 Brazil

## Abstract

**Background:**

The genetic correlation between purebred (PB) and crossbred (CB) performances (${r}_{pc}$) partially determines the response in CB when selection is on PB performance in the parental lines. An earlier study has derived expressions for an upper and lower bound of ${r}_{pc}$, using the variance components of the parental purebred lines, including e.g. the additive genetic variance in the sire line for the trait expressed in one of the dam lines. How to estimate these variance components is not obvious, because animals from one parental line do not have phenotypes for the trait expressed in the other line. Thus, the aim of this study was to propose and compare three methods for approximating the required variance components. The first two methods are based on (co)variances of genomic estimated breeding values (GEBV) in the line of interest, either accounting for shrinkage (VC_GEBV-S_) or not (VC_GEBV_). The third method uses restricted maximum likelihood (REML) estimates directly from univariate and bivariate analyses (VC_REML_) by ignoring that the variance components should refer to the line of interest, rather than to the line in which the trait is expressed. We validated these methods by comparing the resulting predicted bounds of ${r}_{pc}$ with the ${r}_{pc}$ estimated from PB and CB data for five traits in a three-way cross in pigs.

**Results:**

With both VC_GEBV_ and VC_REML_, the estimated ${r}_{pc}$ (plus or minus one standard error) was between the upper and lower bounds in 14 out of 15 cases. However, the range between the bounds was much smaller with VC_REML_ (0.15–0.22) than with VC_GEBV_ (0.44–0.57). With VC_GEBV-S_, the estimated ${r}_{pc}$ was between the upper and lower bounds in only six out of 15 cases, with the bounds ranging from 0.21 to 0.44.

**Conclusions:**

We conclude that using REML estimates of variance components within and between parental lines to predict the bounds of ${r}_{pc}$ resulted in better predictions than methods based on GEBV. Thus, we recommend that the studies that estimate ${r}_{pc}$ with genotype data also report estimated genetic variance components within and between the parental lines.

**Supplementary Information:**

The online version contains supplementary material available at 10.1186/s12711-022-00773-z.

## Background

The genetic correlation between purebred (PB) and crossbred (CB) performances (${r}_{pc}$) is an important parameter in crossbreeding programs, because the ${r}_{pc}$ partially determines the response in CB performance when selection is based on PB performance in the parental lines [1, 2]. Thus; low values of ${r}_{pc}$ may indicate that CB information should be collected when the aim is to improve CB performance.

In livestock populations where crossbreeding is applied, ${r}_{pc}$ is typically lower than 1. For example, the average estimated ${r}_{pc}$ across traits was about 0.63 in pigs [3], and about 0.71 in poultry [4]. Two phenomena can lead to ${r}_{pc}$ values being lower than 1, namely genotype-by-environment interactions (GxE) and genotype-by-genotype interactions (GxG, i.e. non-additive genetic effects).

${r}_{pc}$ is defined as the correlation between the additive genetic values of PB individuals, for PB and CB performances [2]. CB performance can be seen as a trait that is not expressed in the PB breeding individuals themselves, but in their CB offspring. The ${r}_{pc}$ in a given breeding line (e.g. line 1) when mated to another breeding line is defined as:

1 $${r}_{pc}=\frac{{\sigma }_{{1,1}(C)}}{{\sigma }_{1}{\sigma }_{1(C)}},$$

where ${\sigma }_{1}$ is the standard deviation of the additive genetic values for PB performance of individuals in line 1, ${\sigma }_{1\left(C\right)}$ is the standard deviation of the additive genetic values of individuals in line 1 for the trait expressed in CB, and ${\sigma }_{{1,1}\left(C\right)}$ is the covariance between the additive genetic values in line 1 for PB and CB performances [2, 5]. Suppose the causal loci and their true average effects of allele substitution (hereafter called average effects) are known, the vector of additive genetic values of individuals in line 1 for the trait expressed in CB animals (${\mathbf{u}}_{1\left(C\right)}$) could be obtained as the product of the matrix of genotypes of individuals in line 1, and the vector of average effects expressed in CB animals. ${\sigma }_{1\left(C\right)}$ could then be computed as the standard deviation of ${\mathbf{u}}_{1\left(C\right)}$, and ${\sigma }_{{1,1}\left(C\right)}$ as the covariance between ${\mathbf{u}}_{1\left(C\right)}$ and the vector of additive genetic values of individuals in line 1 for the trait expressed in line 1 (i.e. for PB performance, ${\mathbf{u}}_{1}$).

${r}_{pc}$ can be estimated from phenotypes measured on both PB and CB animals when the pedigree relationships between these animals are known. However, in practice collecting CB phenotypes can be costly, and the pedigree of CB animals is often not recorded. The need for pedigree records can be alleviated by genotyping the PB and CB animals that have phenotypes. The genotypes provide a link between PB and CB performances, enabling the estimation of ${r}_{pc}$. Although this approach can lead to accurate estimates of ${r}_{pc}$ [6–8], it requires large investments for phenotyping and genotyping CB animals, while it is uncertain that using such data yields more profit. Therefore it may be beneficial for breeders to use predictions of ${r}_{pc}$ based only on information from the parental lines.

In a previous study, Duenk et al. [9] derived expressions for the approximate bounds of ${r}_{pc}$ based on true variance components computed from the effects of quantitative trait loci (QTL) and the genotypes in the parental lines. These expressions predict the lower and upper bounds of ${r}_{pc}$ when only GxG and no GxE interaction is present. For brevity, we will simply use the symbol ${r}_{pc}$, but this symbol should be interpreted as the decrease of ${r}_{pc}$ due to GxG interactions, throughout the manuscript. The expressions for the bounds of ${r}_{pc}$ allow to predict ${r}_{pc}$ without the use of data from CB animals. For example, the approximate lower bound of ${r}_{pc}$ in line 1 can be predicted as:

2 $${r}_{pc}^{L}=\frac{{\sigma }_{{1,1}(2)}}{{\sigma }_{1}{\sigma }_{1(2)}}.$$

In Eq. (2), ${\sigma }_{1}$ is the standard deviation of the additive genetic values for PB performance in line 1 that is typically estimated from data used in routine evaluations. However, the other two variance components in Eq. (2) differ from the genetic parameters that are typically estimated. ${\sigma }_{1\left(2\right)}$ is the standard deviation of the additive genetic values of individuals in line 1 (i.e., the line for which ${r}_{pc}$ is predicted, called the *focal line*), for the trait expressed in line 2 (called the *trait line*). Suppose the causal loci and their true average effects are known, the vector of additive genetic values of individuals in line 1 for the trait expressed in line 2 (${\mathbf{u}}_{1\left(2\right)}$) could be obtained as the product of the matrix of genotypes of individuals in line 1, and the vector of average effects expressed in line 2. ${\sigma }_{1\left(2\right)}$ could then be computed as the standard deviation of ${\mathbf{u}}_{1\left(2\right)}$, and ${\sigma }_{{1,1}\left(2\right)}$ as the covariance between ${\mathbf{u}}_{1\left(2\right)}$ and ${\mathbf{u}}_{1}$. In other words, ${\sigma }_{1\left(2\right)}$ is a function of the allele frequencies in line 1 and of the average effects for PB performance in line 2. For example, for a single locus, ${\sigma }_{1\left(2\right)}$ would be the square root of $2{p}_{1}\left(1-{p}_{1}\right){\alpha }_{2}^{2}$, where ${p}_{1}$ is the allele frequency in line 1, and ${\alpha }_{2}$ is the average effect in line 2. Similarly, ${\sigma }_{{1,1}\left(2\right)}$ is a function of the allele frequencies in line 1 and of the average effects for PB performance in line 1 and line 2. Although obtaining these two variance components is straightforward when QTL effects and genotypes in the parental lines are known [9], this is not the case when estimates need to be obtained from phenotypes and marker genotypes in the parental lines.

In this study, we propose and compare three methods for approximating the required variance components to predict the bounds of ${r}_{pc}$. The first two methods are based on the variances of and covariances between genomic estimated breeding values (GEBV), assuming that GEBV are useful proxies for true additive genetic values. The third method is based on REML estimates of the variances within and covariances between the parental lines, assuming that the variance of the genotypes (i.e. $2p(1-p)$) across lines is similar. We assess the performance of these three methods by comparing predicted bounds with the estimated value of ${r}_{pc}$ for five traits using data on a three-way cross in pigs.

## Methods

### Dataset

We used phenotypic and genotypic data from three PB lines and their three-way terminal CB, provided by Topigs Norsvin and Norsvin. Sires from a Landrace line (LR) were mated to dams from a Large White line (LW) to produce F1 sows, which were crossed with sires from a synthetic boar line (S) to produce the three-way CB (i.e. Sx(LRxLW)). In total, we used 17,100 animals from line S, 6611 animals from line LR, 8587 animals from line LW, and 4173 three-way CB. The synthetic boar line S is a combination of the LW and Piétrain breeds and was created around 1975 [10]. All purebreds were housed in a nucleus environment, and the crossbreds in a commercial environment, possibly giving rise to GxE interaction between PB and CB performances. Phenotypes were pre-corrected for fixed effects and common litter effects using a larger dataset during the routine genetic evaluation of Topigs Norsvin. The traits included average daily gain during the test period between 30 and 120 kg (TGR), lifetime daily gain (LGR, from birth until 120 kg), daily feed intake during the test period (DFI), backfat (BFE) and loin depth (LDE). In the PB animals, BFE and LDE were measured at the end of the test period using an ultrasound device. In the CB animals, BFE and LDE were measured on the carcass after slaughter at the slaughterhouse.

About 80% of the animals were genotyped with the Illumina 50K single nucleotide polymorphism (SNP) chip and the remaining 20% were genotyped with a custom Illumina 25K SNP chip. All genotypes were imputed to 50K within each purebred line using the FImpute v2.2 software [11]. First, the genotypes were imputed within the purebred lines. Second, the imputed genotypes from all purebred lines were used as a reference population to impute the genotypes of crossbred animals. For quality control, we excluded SNPs with a GenCall rate lower than 0.15 (Illumina Inc., 2005) or a call rate lower than 0.95, SNPs that showed a strong deviation from Hardy–Weinberg equilibrium (χ^2^ > 600), SNPs that were located on sex chromosomes, and unmapped SNPs. The positions of the SNPs were based on the Sscrofa11.1 assembly. Finally, SNPs with a minor allele frequency (MAF) lower than 0.01 in any of the lines were excluded, yielding 35,595 SNPs that were used in all the analyses. All genotyped animals had a frequency of missing genotypes lower than 0.05 and were therefore retained for further analyses.

### Derived expressions for the bounds of ${r}_{pc}$

In a previous study, Duenk et al. [9] derived expressions that predict ${r}_{pc}$ from the genetic variance components of the purebred parental lines. They focused on predicting the decrease of ${r}_{pc}$ due to non-additive effects in a two-way cross, considering two scenarios (genetic models). The first genetic model assumed that there were only additive and dominance genetic effects, and the second genetic model assumed that there were only additive and additive-by-additive (AxA) epistatic genetic effects. The first step was to express the average effects for CB performance (${\alpha }_{CB}$) in the parental line of interest (i.e. the *focal line*) in terms of average effects for PB performance in the parental lines, because the difference between these average effects determines the value of ${r}_{pc}$ [2, 9]. The results showed that for the dominance model, ${\alpha }_{CB}$ in the focal line is equal to the average effect for PB performance in the mated line. As a result, the dominance model may represent a lower bound for ${r}_{pc}$, because the maximum difference between the average effects for PB and CB performances is then bounded by the difference in allele frequencies between the parental lines. For the AxA epistatic model, ${\alpha }_{CB}$ in the focal line is equal to the mean of the average effects for PB performance in both parental lines. As a result, the epistatic model may represent an upper bound of ${r}_{pc}$, because the minimum difference between the average effects for PB and CB performances is bounded by the difference in allele frequencies between the parental line and the cross. Duenk et al. [9] showed that the derived expressions indeed indicate approximate lower and upper bounds of ${r}_{pc}$, regardless of the actual genetic model.

### Predicting bounds of ${r}_{pc}$

The present study builds on the work of Duenk et al. [9] and aims at validating the derived equations for the bounds of ${r}_{pc}$ in real data by evaluating whether the estimated ${r}_{pc}$ falls between the predicted bounds. We predicted the bounds of ${r}_{pc}$ for each parental line based on information from all parental lines, following the expressions for the lower and upper bounds of ${r}_{pc}$ in a three-way cross derived by Duenk et al. [9]. These equations were derived by expressing the additive genetic values of the different groups of animals as products of genotype matrices with vectors of average effects in the parental lines, to allow re-writing the variance components that appear in Eq. (1) in terms of variance components in the PB lines. Full derivations are presented in the appendix of Duenk et al. [9]. The predicted lower bound of ${r}_{pc}$ in sire line S for the three-way CB is:

3 $${r}_{pc,S}^{L}=\frac{{\sigma }_{S(S),S\left(LW\right)}+{\sigma }_{S(S),S\left(LR\right)}}{{\sigma }_{S}\sqrt{\left({\sigma }_{S\left(LW\right)}^{2}+{\sigma }_{S\left(LR\right)}^{2}+2{\sigma }_{S\left(LW\right),S\left(LR\right)}\right)}},$$

where ${\sigma }_{S}$ is the standard deviation of the additive genetic values of individuals in line S for the trait expressed in line S, ${\sigma }_{S\left(LW\right)}^{2}$ and ${\sigma }_{S\left(LR\right)}^{2}$ are the variance of the additive genetic values of individuals in line S for the trait expressed in line LW or LR, respectively, and ${\sigma }_{S\left(LW\right),S\left(LR\right)}$ is the covariance between the additive genetic values of individuals in line S for the trait expressed in line LW and LR. For across-line variance components such as ${\sigma }_{S\left(LW\right)}^{2}$, we use the term *focal line* to refer to the line for which ${r}_{pc}$ is predicted (S), and the term *trait line* to refer to the line where the trait is expressed (LW). The predicted upper bound of ${r}_{pc}$ in sire line S uses the same parameters and is:

4 $${r}_{pc,S}^{U}=\frac{{\sigma }_{S}^{2}+0.5{\sigma }_{S(S),S\left(LW\right)}+0.5{\sigma }_{S(S),S\left(LR\right)}}{{\sigma }_{S}\sqrt{\left({\sigma }_{S}^{2}+0.25{\sigma }_{S(LW)}^{2}+0.25{\sigma }_{S(LR)}^{2}+{\sigma }_{S(S),S\left(LW\right)}+{\sigma }_{S(S),S\left(LR\right)}+0.5{\sigma }_{S(LW),S\left(LR\right)}\right)}},$$

where the parameters are the same as those in Eq. (3).The bounds of ${r}_{pc}$ in lines LW and LR were predicted in a similar way, but using expressions for dam lines of a three-way CB. The predicted lower bound of ${r}_{pc}$ in dam line LW is:

5 $${r}_{pc,LW}^{L}=\frac{{\sigma }_{LW(LW),LW(S)}}{{\sigma }_{LW}{\sigma }_{LW(S)}},$$

where ${\sigma }_{LW}$ is the standard deviation of the additive genetic values of individuals in line LW for the trait expressed in line LW, ${\sigma }_{LW\left(S\right)}^{2}$ is the variance of the additive genetic values of individuals in line LW for the trait expressed in line S, and ${\sigma }_{LW\left(LW\right),LW\left(S\right)}$ is the covariance between the additive genetic values of individuals in line LW for the trait expressed in line LW and S. The predicted upper bound of ${r}_{pc}$ in dam line LW is:

6 $${r}_{pc,LW}^{U}=\frac{{\sigma }_{LW\left(LW\right), LW\left(S\right)}+0.5{\sigma }_{LW}^{2}+0.5{\sigma }_{LW(LW),LW(LR)}}{{\sigma }_{LW}\sqrt{\left({\sigma }_{LW(S)}^{2}+0.25{\sigma }_{LW}^{2}+0.25{\sigma }_{LW\left(LR\right)}^{2}+{\sigma }_{LW\left(LW\right), LW\left(S\right)}+{\sigma }_{LW\left(S\right),LW(LR)}+0.5{\sigma }_{LW(LW),LW(LR)}\right)}},$$

where ${\sigma }_{LW\left(LR\right)}^{2}$ is the variance of the additive genetic values of individuals in line LW for the trait expressed in line LR, ${\sigma }_{LW\left(S\right),LW\left(LR\right)}$ is the covariance between the additive genetic values of individuals in line LW for the trait expressed in line S and LR, ${\sigma }_{LW\left(LW\right),LW\left(LR\right)}$ is the covariance between the additive genetic values of individuals in line LW for the trait expressed in line LW and LR, and the other parameters are the same as those in Eq. (5). The expression for the lower and upper bounds of ${r}_{pc}$ in dam line LR are obtained by replacing subscript LW with LR, and vice versa, in Eqs. (5) and (6).

### Approximating variance components

Equations (3) to (6) show that we need the genetic variance components of the parental lines, which are not usually available, such as the additive genetic standard deviation in individuals of line S, for the trait expressed in line LW (${\sigma }_{S\left(LW\right)}$). These across-line variance component cannot be estimated directly, because there are no phenotypes of the individuals from line S for the trait expressed in line LW. Therefore, here we compare three methods for approximating such variance components.

The first method (VC_GEBV_) relies on the assumption that the GEBV are estimates of true additive genetic values. Thus, the GEBV may be used as proxies for the additive genetic values in the focal line, and the required variance components can be approximated by computing the covariances between these GEBV and their variances. A disadvantage of VC_GEBV_ is that the variance of GEBV is expected to be smaller than the variance of the additive genetic values (${\sigma }_{a}^{2}$) due to shrinkage (i.e. the variance of GEBV is equal to ${r}^{2}{\sigma }_{a}^{2}$, where ${r}^{2}$ is the reliability of GEBV), resulting in biased estimates of variance components. Thus, we included a second method (VC_GEBV-S_), where the GEBV were corrected for shrinkage by dividing them by the square root of their reliabilities.

The third method for approximating variance components was based on REML estimates (VC_REML_). It uses estimates of genetic variances resulting from a univariate analysis of the trait line, and estimates of genetic covariances resulting from pairwise bivariate analyses between parental lines. On the one hand, this method ignores that the required across-line variance components should correspond to individuals in the focal line, and instead uses the components estimated in the trait line. The across-line variance in the focal line can differ from the variance in the trait line due to differences in genotype frequencies between lines. On the other hand, REML estimates are generally accurate, and commonly reported. Furthermore, this method may result in accurate approximations when the genotype frequencies in the focal line are similar to those in the trait line.

Note that, for each of these methods, all variance components in Eqs. (3) to (6) were approximated using the method described, including the within-line variance components such as ${\sigma }_{S}$.

#### Methods based on GEBV (VC_GEBV_ and VC_GEBV__-S_)

With VC_GEBV,_ the required variance components are approximated by using the GEBV as proxies for true additive genetic values. We first analysed phenotypes in each PB line separately using a univariate model, while including genotypes of all PB lines to obtain GEBV for all PB animals. For example, for the phenotypes in line S, the model was:

7 $${\mathbf{y}}_{S}={\mu }_{S}+\mathbf{Z}{\mathbf{u}}_{S}+{\mathbf{e}}_{S},$$

where ${\mathbf{y}}_{S}$ is the vector of corrected phenotypes, ${\mu }_{S}$ is the mean, ${\mathbf{u}}_{S}$ is the vector of additive genetic values with incidence matrix $\mathbf{Z}$, and $\mathbf{e}$ is the vector of independent random residuals. The additive genetic values were distributed as ${\mathbf{u}}_{S} \sim N\left(\mathbf{0},{\sigma }_{s}^{2}\mathbf{G}\right)$, where ${\sigma }_{s}^{2}$ is the additive genetic variance in line S, and $\mathbf{G}$ is the multi-breed genomic relationship matrix that includes all PB animals and is constructed following Wientjes et al. [12]. Note that this approach is equivalent to estimating marker effects in line S, and then multiplying these estimates with marker genotypes of all PB animals [13, 14]. The model results in GEBV for all individuals from lines S, LW, and LR, for the trait expressed in line S (${\widehat{\mathbf{u}}}_{All(S)}$). The same approach was used to estimate GEBV of all PB animals for the trait expressed in lines LW (${\widehat{\mathbf{u}}}_{All(LW)}$) and LR (${\widehat{\mathbf{u}}}_{All(LR)}$), using the phenotypes of those lines.

The variances that appear in Eqs. (3) to (6) were approximated as the standard deviations of GEBV of individuals in the corresponding focal line. For example, ${\sigma }_{LW\left(S\right)}$ was approximated as $\sqrt{var\left({\widehat{\mathbf{u}}}_{LW\left(S\right)}\right)}$, where ${\widehat{\mathbf{u}}}_{LW(S)}$ is a subset of ${\widehat{\mathbf{u}}}_{All(S)}$. The covariances that appear in Eqs. (3) to (6) were approximated as the covariance between GEBV of individuals in the focal line. For example, ${\sigma }_{LW\left(S\right), LW\left(LR\right)}$ was approximated as $cov\left({\widehat{\mathbf{u}}}_{LW\left(S\right)}, {\widehat{\mathbf{u}}}_{LW\left(LR\right)}\right)$, where ${\widehat{\mathbf{u}}}_{LW\left(S\right)}$ and ${\widehat{\mathbf{u}}}_{LW\left(LR\right)}$ are the GEBV of individuals in line LW, for the trait expressed in line S and line LR, respectively. The advantage of VC_GEBV_ is that the resulting variance components relate correctly to the individuals in the focal line.

Variances and covariances of GEBV can be poor approximations of genetic variance components, because GEBV are subject to shrinkage. For example, the variance of GEBV is a factor ${r}^{2}$ of the corresponding additive genetic variance, where ${r}^{2}$ is the reliability of the GEBV [15]. Therefore, with the second method (VC_GEBV-S_), we used the same approach as VC_GEBV_, but we divided the GEBV by the square-root of their individual reliabilities to correct for shrinkage. The individual reliabilities were computed by the MTG2 program [16], using the prediction error variance [17]. This procedure aims at scaling the GEBV such that their variance is independent of their reliabilities. The variances of and the covariances between the scaled GEBV were used to approximate the parameters in Eqs. (3) to (6). Note that this approach is similar to the approach of Calo et al. [18] and Blanchard et al. [19], except that here we perform scaling at the level of the GEBV while allowing for different individual reliabilities, whereas in [18] and Blanchard et al. [19] scaling is performed at the level of the variances and covariances using the sum of the reliabilities across individuals.

#### Method based on REML estimates (VC_REML_)

With the third method (VC_REML_), we used genomic REML estimates of variance components within and between lines to approximate the required parameters. Approximated variances and standard deviations resulted from univariate genomic analyses in the trait lines. For example, ${\sigma }_{S\left(LW\right)}$ and ${\sigma }_{LR\left(LW\right)}$ were approximated as the REML estimate of the additive genetic standard deviation in line LW (${\sigma }_{LW}$). The model used for this analysis was:

8 $${\mathbf{y}}_{LW}={\mu }_{LW}+\mathbf{Z}{\mathbf{u}}_{LW}+{\mathbf{e}}_{LW},$$

where ${\mathbf{y}}_{LW}$ is the vector of corrected phenotypes in line LW, ${\mu }_{LW}$ is the mean, ${\mathbf{u}}_{LW}$ is the vector of additive genetic values with incidence matrix $\mathbf{Z}$, and ${\mathbf{e}}_{LW}$ is the vector of independent random residuals. The additive genetic values were distributed as ${\mathbf{u}}_{LW} \sim N\left(\mathbf{0},{\sigma }_{LW}^{2}{\mathbf{G}}_{LW}\right)$, where ${\sigma }_{LW}^{2}$ is the additive genetic variance in line LW, ${\mathbf{G}}_{LW}$ is the genomic relationship matrix and is constructed following method 2 of VanRaden [20]. The REML estimate ${\widehat{\sigma }}_{LW}$ is used as an approximation for the standard deviation of the additive genetic values for the trait expressed in line LW (i.e. ${\sigma }_{LW}$, ${\sigma }_{S\left(LW\right)}$ and ${\sigma }_{LR\left(LW\right)}$). The same approach was used to approximate variance components for the trait expressed in lines S (${\sigma }_{S}$, ${\sigma }_{LW(S)}$ and ${\sigma }_{LR(S)}$) and LR (${\sigma }_{LR}, {\sigma }_{S(LR)}$ and ${\sigma }_{LW(LR)}$), using data of those lines.

Approximate covariances resulted from pairwise bivariate genomic analyses for each combination of two parental lines. For example, the covariance ${\sigma }_{S\left(LW\right),S(LR)}$ was approximated as the REML estimate of the additive genetic covariance between lines LW and LR (${\sigma }_{LW,LR}$), resulting from the bivariate analysis of performance in lines LW and LR. The model can be written as [21, 22]:

9 $$\left[\begin{array}{c}{\mathbf{y}}_{LW}\\ {\mathbf{y}}_{LR}\end{array}\right] =\left[\begin{array}{cc}{\mathbf{1}}_{LW}& {\mathbf{0}} \\ {\mathbf{0}} & {\mathbf{1}}_{LR}\end{array}\right]\left[\begin{array}{c}{\mu }_{LW}\\ {\mu }_{LR}\end{array}\right]+\left[\begin{array}{cc}{\mathbf{Z}}_{LW} & 0\\ 0& {\mathbf{Z}}_{LR}\end{array}\right]\left[\begin{array}{c}{\mathbf{u}}_{LW}\\ {\mathbf{u}}_{LR}\end{array}\right]+\left[\begin{array}{c}{\mathbf{e}}_{LW}\\ {\mathbf{e}}_{LR}\end{array}\right],$$

where $\mathbf{y}$ is the vector of corrected phenotypes, $\mu$ is the mean, $\mathbf{1}$ is a column vector of 1s, $\mathbf{u}$ is the vector of additive genetic values with incidence matrix $\mathbf{Z}$, and $\mathbf{e}$ is the vector of independent random residuals. Subscripts denote whether the terms relate to LW or LR animals. The distribution of the additive genetic values was:

10 $$\left[\begin{array}{c}{\mathbf{u}}_{LW}\\ {\mathbf{u}}_{LR}\end{array}\right]\sim N\left(\left[\begin{array}{c} {\mathbf{0}} \\ {\mathbf{0}} \end{array}\right], \left[\begin{array}{cc}{\sigma }_{LW}^{2}& {\sigma }_{LW,LR}\\ {\sigma }_{LW,LR}& {\sigma }_{LR}^{2}\end{array}\right]\otimes \mathbf{G}\right),$$

where ${\sigma }_{LW}^{2}$ is the additive genetic variance in line LW, ${\sigma }_{LR}^{2}$ is the additive genetic variance in line LR, and ${\sigma }_{LW,LR}$ is the additive genetic covariance between lines LW and LR. The genomic relationship matrix between all individuals ($\mathbf{G}$) was calculated following Wientjes et al. [12]. The variance components were estimated using REML in the MTG2 software [16]$.$ The same approach was used to estimate covariances for each combination of two parental lines.

In addition to REML estimates of genetic variances and covariances, the bivariate analyses resulted in estimates of genetic correlations between parental lines (computed as ${\widehat{r}}_{g}=\frac{{\widehat{\sigma }}_{{1,2}}}{{\widehat{\sigma }}_{1}{\widehat{\sigma }}_{2}}$). We report these genetic correlations in the Results section because they are related to (the predicted bounds of) ${r}_{pc}$. Both ${r}_{g}$ and ${r}_{pc}$ depend on the size of the non-additive genetic effects and the differences in allele frequencies between parental lines [9, 23].

### Estimating ${r}_{pc}$ and validation of predicted bounds

We estimated ${r}_{pc}$ for each of the parental lines using a bivariate model that treats PB and CB performances as different, but genetically correlated traits. Note that we did not combine data from all PB lines in a single analysis, but we estimated ${r}_{pc}$ for each parental line, separately, using three bivariate models. The statistical model was similar to the bivariate model that was used to estimate REML estimates of covariances between lines (Eq. (9)), except that the data used were from PB and CB animals, instead of from lines LW and LR. The variance components were estimated using REML in the MTG2 software [16], and ${r}_{pc}$ was estimated as ${\widehat{r}}_{pc}=\frac{{\widehat{\sigma }}_{PB,CB}}{{\widehat{\sigma }}_{PB}{\widehat{\sigma }}_{CB}}.$

We validated the predicted bounds by comparing them with the estimated ${r}_{pc}.$ Note that, in these comparisons, we consider the predicted bounds to be correct if the interval of the estimated ${r}_{pc}$ plus and minus one standard error of the estimate overlapped with the interval between the predicted bounds. Although the predicted bounds may capture the estimated ${r}_{pc}$, the bounds may not be very informative if they cover an extremely large range. For example, a predicted lower bound of − 1 and a predicted upper bound of 1 will always capture estimated ${r}_{pc}$, but such a prediction is not useful at all. Thus, we computed the range of bounds as the difference between the predicted upper and lower bounds of ${r}_{pc}$.

## Results

First, we compare the predicted bounds of ${r}_{pc}$ with the estimated ${r}_{pc}$. Second, we present the results on estimated genetic parameters such as heritabilities, ${r}_{pc},$ and genetic correlations between lines.

### Predicted bounds of ${r}_{pc}$

**Fig. 1:**
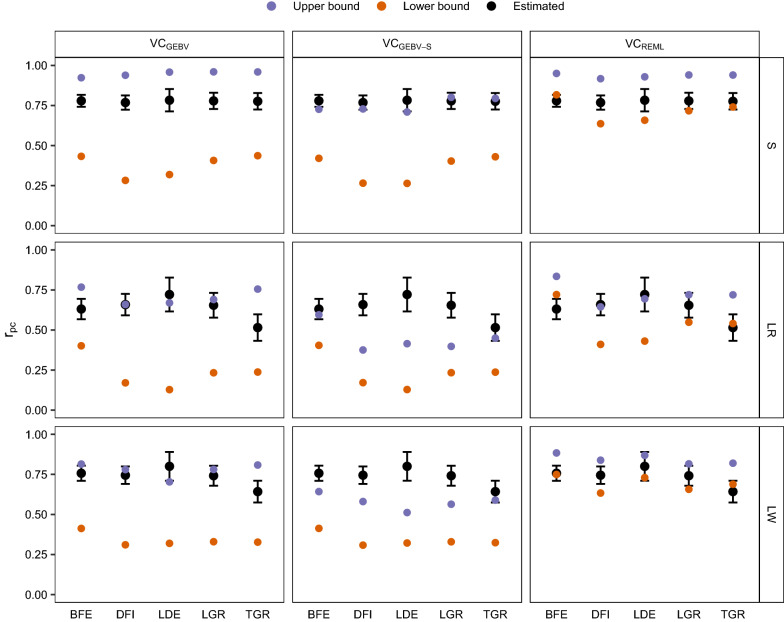
Estimated ${r}_{pc}$ and predicted bounds of ${r}_{pc}$ (y-axis) for all traits (x-axis). Whiskers indicate the standard error reported by the MTG2 program. Column facets indicate the method that was used to approximate variance components, and row facets indicate the focal line. *BFE* backfat, *DFI* daily feed intake, *LDE* loin depth, *LGR* lifetime daily gain, *TGR* test growth rate, *S* synthetic boar line, *LR* Landrace line, *LW* Large-White line

Of the 15 trait-line combinations, the estimated ${r}_{pc}$ (plus or minus one standard error) fell between the predicted lower and upper bounds in 14 cases with VC_GEBV_, 6 cases with VC_GEBV-S_, and 14 cases with VC_REML_ (Fig. 1). With methods based on GEBV, the estimated ${r}_{pc}$ was usually closer to the upper bound than to the lower bound, while for VC_REML_, this varied across traits and lines.

On average across traits, VC_GEBV_ resulted in the largest range for all lines (0.44–0.57), followed by VC_GEBV-S_ (0.21–0.40) and VC_REML_ (0.15–0.22) (Table 1). With VC_REML_, the average range across traits was largest for line S (0.22), followed by the LR (0.19) and LW (0.15) lines.

**Table 1:** Mean and standard deviations (SD) of the difference between the predicted upper and lower bounds across traits, for three lines (presented in columns) and three methods of predicting bounds (presented in rows)

Method	S	LR	LW
VC_GEBV_	0.57 (0.07)	0.47 (0.07)	0.44 (0.04)
VC_GEBV-S_	0.40 (0.06)	0.21 (0.05)	0.24 (0.03)
VC_REML_	0.22 (0.07)	0.19 (0.06)	0.15 (0.03)

*S* synthetic boar line, *LR* Landrace line, *LW* Large White line

### Estimated genetic parameters

The estimated heritabilities for PB performance across traits ranged from 0.23 (TGR for line S) to 0.54 (BFE for line LW) (Table 2). Across traits, the average heritability was lowest in line S (0.36), followed by lines LR (0.39) and LW (0.44). Across lines, the average heritability was lowest for TGR (0.32), and highest for BFE (0.48).

**Table 2:** Estimated heritabilities (${h}^{2}$) and their standard errors (between brackets) for all traits (presented in columns), and three PB parental lines and their CB (presented in rows)

Line	BFE	DFI	LDE	LGR	TGR
S	0.51 (0.01)	0.35 (0.01)	0.46 (0.01)	0.26 (0.01)	0.23 (0.01)
LR	0.41 (0.02)	0.49 (0.02)	0.32 (0.02)	0.39 (0.02)	0.36 (0.02)
LW	0.54 (0.01)	0.48 (0.02)	0.45 (0.02)	0.38 (0.02)	0.35 (0.02)
CB	0.31 (0.02)	0.26 (0.02)	0.10 (0.02)	0.22 (0.02)	0.24 (0.02)

*BFE* backfat thickness, *DFI* daily feed intake, *LDE* loin depth, *LGR* lifetime daily gain, *TGR* test growth, *S* synthetic boar line, *LR* Landrace line, *LW* Large White line, *CB* three-way crossbreds

The estimated ${r}_{pc}$ across traits and lines ranged from 0.52 (TGR in line LR) to 0.80 (LDE in line LW), with an average of 0.72 (Table 3). On average across traits, the estimated ${r}_{pc}$ was highest in line S (0.78), followed by lines LW (0.74) and LR (0.64). On average across lines, the estimated ${r}_{pc}$ was lowest for TGR (0.65) and highest for LDE (0.77). The standard errors across traits and lines ranged from 0.04 (DFI and BFE in line S) to 0.11 (LDE in line LR), with an average of 0.06. On average, estimated ${r}_{pc}$ in line S had the smallest standard errors (0.05), followed by lines LW (0.06) and LR (0.08).

**Table 3:** Estimated correlation between purebred and crossbred performances (${r}_{pc}$) and their standard errors (between brackets) for all traits (indicated in columns) and three PB parental lines (presented in rows)

Line	BFE	DFI	LDE	LGR	TGR
S	0.78 (0.04)	0.77 (0.04)	0.78 (0.07)	0.78 (0.05)	0.78 (0.05)
LR	0.63 (0.06)	0.66 (0.07)	0.72 (0.11)	0.65 (0.08)	0.52 (0.08)
LW	0.76 (0.05)	0.74 (0.05)	0.80 (0.09)	0.74 (0.06)	0.64 (0.07)

*BFE* backfat thickness, *DFI* daily feed intake, *LDE* loin depth, *LGR* lifetime daily gain, *TGR* test growth, *S* synthetic boar line, *LR* Landrace line, *LW* Large White line

The estimated genetic correlation between lines (${r}_{g}$) across traits and line combinations ranged from 0.39 (TGR for LR-LW) to 0.75 (BFE for S-LW) (Table 4). On average across traits, the estimated ${r}_{g}$ was lowest between lines LR and LW (0.48), followed by S-LR (0.53) and S-LW (0.69). On average across lines, the estimated ${r}_{g}$ was lowest for DFI (0.49), and highest for BFE (0.70). The standard errors across traits and lines ranged from 0.03 (BFE for S-LW) to 0.08 (LDE, LGR and TGR for LR-LW), with an average of 0.06.

**Table 4:** Estimated genetic correlations (${r}_{g}$) between PB parental lines (presented in rows) and their standard errors (between brackets) for all traits (presented in columns)

	BFE	DFI	LDE	LGR	TGR
S-LR	0.72 (0.06)	0.41 (0.07)	0.43 (0.07)	0.55 (0.07)	0.54 (0.08)
LW-S	0.75 (0.03)	0.63 (0.04)	0.72 (0.04)	0.66 (0.05)	0.69 (0.05)
LW-LR	0.62 (0.06)	0.44 (0.07)	0.54 (0.08)	0.43 (0.08)	0.39 (0.08)

*BFE* backfat thickness, *DFI* daily feed intake, *LDE* loin depth, *LGR* lifetime daily gain, *TGR* test growth, *S* synthetic boar line, *LR* Landrace line, *LW* Large White line

## Discussion

The objective of this study was to empirically validate expressions that predict the bounds of ${r}_{pc}$ based on variance components of the parental lines, without requiring CB data. Our results showed that it is indeed possible to predict the bounds of ${r}_{pc}$, and that REML estimates provide useful approximations for the required variance components.

### Comparison of methods

#### Methods based on GEBV (VC_GEBV_ and VC_GEBV__-S_)

The first method to predict the bounds of ${r}_{pc}$ was based on the variances of and covariances between GEBV of individuals in the focal line (VC_GEBV_). Compared to the other methods we proposed, the advantage of VC_GEBV_ is that the resulting variance components correspond to the individuals in the focal line, and not to the mated lines. Our results showed that VC_GEBV_ resulted in a high fraction of cases where the predicted bounds captured the estimated ${r}_{pc}$, but that the range between the lower and upper bounds was relatively large.

A disadvantage of VC_GEBV_ is that the variance of GEBV is expected to be smaller than the variance of additive genetic values due to shrinkage, resulting in biased estimates of variance components. Thus, we included a second method (VC_GEBV-S_), where GEBV were corrected for shrinkage by dividing them by the square root of their reliabilities. Although VC_GEBV-S_ generally resulted in a smaller range than VC_GEBV_, it also resulted in a high fraction of cases where the estimated ${r}_{pc}$ was higher than the predicted upper bound, especially in lines LR and LW.

#### Method based on REML (VC_REML_)

The third method for approximating variance components was based on REML estimates (VC_REML_). Our results show that, across traits and lines, VC_REML_ resulted in more accurate prediction of the bounds of ${r}_{pc}$ than VC_GEBV_ and VC_GEBV-S_ because it resulted in the highest fraction of cases (14 out of 15) where the predicted bounds captured the estimated ${r}_{pc}$, and the smallest range between the lower and upper bounds. This result was somewhat surprising, because the across-line variance components from VC_REML_ do not correspond to the individuals of the focal line, but to those of the trait line (i.e., the mated lines from which phenotypes were analysed). We therefore investigated whether the within-line variance of the trait line was comparable to the across-line variance of the focal line. The within-line variance in the trait line is proportional to heterozygosity ($\sum 2{p}_{i}\left(1-{p}_{i}\right)$) in that line [5]. In contrast, the variance in the focal line for the trait expressed in the other line is proportional to the heterozygosity in the focal line. In this study, the difference in heterozygosity between parental lines was small, although estimated additive genetic variances differed considerably (see Additional file 1: Table S1). This similarity suggests that the across-line variances in the focal lines are similar to the corresponding within-line variances in the trait lines, and that therefore VC_REML_ results in relatively accurate approximations of across-line variance components. Thus, we conclude that, when the heterozygosities in the parental lines are comparable, the bounds of ${r}_{pc}$ can be relatively well predicted from REML estimates of the genetic variances within and the covariances between parental lines.

For other combinations of PB parental lines, the difference in heterozygosity between lines may be larger than in this study. Larger differences can occur when parental lines differ strongly in their history, for example due to differences in selection or in effective population size, or due to population admixture or migration. In those cases, the VC_REML_ method may be improved by scaling the REML estimates to the focal line. For example, ${\sigma }_{S\left(LW\right)}^{2}$ may be approximated by multiplying the REML estimate of within-line variance in LW (${\sigma }_{LW}^{2}$) with $\sum 2{p}_{S}\left(1-{p}_{S}\right){\left(\sum 2{p}_{LW}\left(1-{p}_{LW}\right) \right)}^{-1}$. Similarly, ${\sigma }_{S,S(LW)}$ may be approximated by multiplying the REML estimate of ${\sigma }_{S,LW}$ by $\sqrt{\sum 2{p}_{S}\left(1-{p}_{S}\right)}{\left(\sqrt{\sum 2{p}_{LW}\left(1-{p}_{LW}\right)}\right)}^{-1}$. However, this scaling procedure may not necessarily affect the predicted bounds, because scaling occurs in both the numerators and denominators of the equations to predict ${r}_{pc}$. To test this effect of scaling, we considered an extreme hypothetical example where the heterozygosity ($\sum 2{p}_{i}\left(1-{p}_{i}\right)$) in three parental lines (A, B, and C) differed greatly: the heterozygosity in line B was twice as large as in line A, and in line C it was four times as large as in line A. In this example, we used the REML estimates of the DFI trait presented in this study to predict the bounds of ${r}_{pc}$ in all parental lines, and compared the predicted bounds when the across-line variance components were either scaled to the focal line, or not. The results showed that scaling resulted in a difference between predicted bounds of at most 0.06 (results not shown). In fact, the predicted lower bound in the dam lines did not change at all, because the scaling is completely cancelled out in the prediction equation. These results suggest that using VC_REML_ may yield useful predictions of the bounds of ${r}_{pc}$, even when the parental lines differ strongly in heterozygosity.

### Estimation of genetic parameters

The estimated ${r}_{pc}$ ranged from 0.52 to 0.80 across lines and traits, which matches with the range reported in Wientjes and Calus [3] and with estimates from recent studies [24–26]. For most traits, ${r}_{pc}$ estimates were higher in line S than in lines LW and LR. This result was expected, because 50% of the CB genome originates from sire line S, whereas only 25% of the CB genome originates from dam lines LW and LR. For all traits, the ${r}_{pc}$ was lowest in line LR, probably because line LR is distantly related to the other lines S and LW, whereas lines S and LW themselves are more closely related.

We estimated ${r}_{pc}$ using a bivariate model that considered PB and CB performances as different, but correlated traits. In this model, genomic relationships between PB and CB animals were computed using all the alleles in the CB animals, ignoring that part of the alleles originated from other PB lines and did not contribute to the relationship with the PB line of interest. Ideally, genomic relationships between PB and CB animals should be computed using only the alleles in CB animals that originated from the PB line of interest, by tracing the breed-of-origin of alleles (BOA) [27]. However, in an empirical study of broiler chicken, estimates of ${r}_{pc}$ from models that ignored or considered the BOA were very similar [6]. The authors argued that, when ignoring the BOA, the alleles from the mated lines do not contribute to variation in relationships between the CB and the parental lines of interest [28, 29]. As a result, the estimation of variance components was dominated by the alleles in CB that originated from the parental line of interest. This result may suggest that considering the BOA in the current study would not have affected the estimates of ${r}_{pc}$.

Across PB lines and traits, the heritability estimates ranged from 0.23 to 0.54, and were generally in line with estimates from recent studies [24–26, 30–32]. It should be noted that the estimated heritabilities reported in this study did not account for the fact that phenotypes were pre-corrected for random common litter effects. The heritability estimates reported here may therefore be somewhat inflated. For all traits, the heritability estimate for CB performance was lower than that for PB performance, which is in line with results from Zumbach et al. [33]. For most traits (DFI, LDE, LGR, and TGR), this difference was due to smaller estimated additive genetic variances for CB than for PB performance (see Additional file 1: Table S1), while for the other traits (BFE and LDE), this difference was due to greater estimated residual variances for CB than for PB performance (see Additional file 1: Table S2).

The estimated genetic correlations between lines (${r}_{g}$) ranged from 0.39 to 0.75 across traits. These estimates indicate that the genes and their effects underlying the studied traits are at least partially the same across parental lines. To our knowledge, genetic correlations between pig breeds have not been previously reported for production traits. The highest genetic correlations were found between lines S and LW, which was expected, because the synthetic boar lines S historically originated from individuals of line LW.

### Practical considerations

Although the prediction of ${r}_{pc}$ with the approach proposed in this study does not require data on CB animals, it does require data on all the PB parental lines that are used to make the commercial cross. In practice, however, phenotypes of certain traits may not be collected routinely in all the parental lines. For example, phenotypes for reproduction traits may not be collected in the sire line. Therefore, for some traits, the methods proposed in this study may require that additional data are collected on the PB parental lines.

The present study shows that the bounds of ${r}_{pc}$ can be predicted based on parental line information, but that the exact value of ${r}_{pc}$ may remain unknown. However, these bounds may indicate whether it is worthwhile to collect data on CB animals to estimate ${r}_{pc}$ and to predict the breeding values for CB performance in the parental lines. For example, if the predicted upper bound of ${r}_{pc}$ is lower than 0.85, it is very likely that the true ${r}_{pc}$ is close to or below that value, in which case collection of CB data is expected to be beneficial [34]. In the case of Xiang et al. [35], the predicted upper bound of ${r}_{pc}$ for total number born based on reported variance components would be 0.84, which suggests that the collection of CB data would be beneficial. The estimated ${r}_{pc}$ in that study (0.67 ± 0.10) was indeed lower than this predicted upper bound.

## Conclusions

In conclusion, using REML estimates of variance components within and between parental lines to predict the bounds of ${r}_{pc}$ resulted in better predictions than methods based on GEBV. If confirmed with other datasets, this approach may help breeders to predict the benefit of collecting CB data based only on parental line information. Thus, we recommend that studies that estimate ${r}_{pc}$ with genotype data also report estimated genetic variance components within and between the parental lines, by estimating them as described in this paper.

## Supplementary Information

**Additional file 1: Table S1.** Estimated additive genetic variances. Description: Sum of variance of marker genotypes (${\sigma }_{x}^{2}$) and estimated additive genetic variances (${\widehat{\sigma }}_{a}^{2}$) with standard errors (se), for each trait (presented in columns), for all parental lines (S, LR, and LW) and their crossbred (CB) (presented in rows). **Table S2.** Estimated residual variances. Description: Estimated residual variances (${\widehat{\sigma }}_{e}^{2}$) with standard errors (se), for each trait (presented in columns), for all parental lines (S, LR, and LW) and their crossbred (CB) (presented in rows).

## Data Availability

The data analysed in this study are not publicly available because they are part of the commercial breeding program of Topigs Norsvin and Norsvin. They are however available upon reasonable request.
