# Supplementary material for: Predicting the impact of genotype-by-genotype interaction on the purebred–crossbred genetic correlation from phenotype and genotype marker data of parental lines
Source: Genet Sel Evol. 2023 Jan 13;55:2. doi: 10.1186/s12711-022-00773-z (PMC9837999; doi:10.1186/s12711-022-00773-z)
Supplement: Supplementary file 1 — Additional file 1: Table S1. Estimated additive genetic variances. Description: Sum of variance of marker genotypes (${\sigma }_{x}^{2}$σx2) and estimated additive genetic variances (${\widehat{\sigma }}_{a}^{2}$σ^a2) with standard errors (se), for each trait (presented in columns), for all parental lines (S, LR, and LW) and their crossbred (CB) (presented in rows). Table S2. Estimated residual variances. Description: Estimated residual variances (${\widehat{\sigma }}_{e}^{2}$σ^e2) with standard errors (se), for each trait (presented in columns), for all parental lines (S, LR, and LW) and their crossbred (CB) (presented in rows). [file 12711_2022_773_MOESM1_ESM.docx]

***Table S1*** *Sum of variance of marker genotypes (*$\sigma_{x}^{2}$*) and estimated additive genetic variances (* $\hat{\sigma}_{a}^{2}$*) with standard errors (se), for each trait (presented in columns), for all parental lines (S, LR, and LW) and their crossbred (CB) (presented in rows).*

|  |  | **BFE** | **DFI** | **LDE** | **LGR** | **TGR** |
| --- | --- | --- | --- | --- | --- | --- |
| line | $\sigma_{x}^{2}$ | $\hat{\sigma}_{a}^{2}$ (se) | $\hat{\sigma}_{a}^{2}$ (se) | $\hat{\sigma}_{a}^{2}$ (se) | $\hat{\sigma}_{a}^{2}$ (se) | $\hat{\sigma}_{a}^{2}$ (se) |
| **S** | 14837 | 1.03 (0.04) | 16539 (806) | 7.47 (0.32) | 920 (51) | 2453 (143) |
| **LR** | 13651 | 1.02 (0.07) | 11662 (685) | 6.75 (0.49) | 709 (47) | 2596 (178) |
| **LW** | 16972 | 1.90 (0.09) | 24479 (1255) | 6.08 (0.33) | 1047 (61) | 3109 (188) |
| **CB** | 18326 | 1.40 (0.13) | 9701 (989) | 3.40 (0.61) | 408 (44) | 1526 (161) |

*BFE=backfat thickness, DFI=daily feed intake, LDE=loin depth, LGR=lifetime daily gain, TGR=test growth. S=synthetic boar line, LR=Landrace line, LW=Large White line, CB = three-way crossbreds.*

***Table S2*** *Estimated residual variances (* $\hat{\sigma}_{e}^{2}$*) with standard errors (se), for each trait (presented in columns), for all parental lines (S, LR, and LW) and their crossbred (CB) (presented in rows).*

|  | **BFE** | **DFI** | **LDE** | **LGR** | **TGR** |
| --- | --- | --- | --- | --- | --- |
| line | $\hat{\sigma}_{e}^{2}$ (se) | $\hat{\sigma}_{e}^{2}$ (se) | $\hat{\sigma}_{e}^{2}$ (se) | $\hat{\sigma}_{e}^{2}$ (se) | $\hat{\sigma}_{e}^{2}$ (se) |
| **S** | 1.00 (0.01) | 30142 (361) | 8.63 (0.11) | 2679 (31) | 8211 (96) |
| **LR** | 1.48 (0.03) | 12377 (266) | 14.36 (0.29) | 1130 (24) | 4576 (95) |
| **LW** | 1.63 (0.03) | 26187 (493) | 7.49 (0.14) | 1683 (31) | 5688 (103) |
| **CB** | 3.10 (0.09) | 27734 (789) | 32.03 (0.82) | 1416 (39) | 4967 (138) |

*BFE=backfat thickness, DFI=daily feed intake, LDE=loin depth, LGR=lifetime daily gain, TGR=test growth. S=synthetic boar line, LR=Landrace line, LW=Large White line, CB = three-way crossbreds.*
